# Optimal cutoff values for anthropometric indices of obesity as discriminators of metabolic abnormalities in Korea: results from a Health Examinees study

**DOI:** 10.1186/s12889-021-10490-9

**Published:** 2021-03-06

**Authors:** Sooyoung Cho, Aesun Shin, Ji-Yeob Choi, Sang Min Park, Daehee Kang, Jong-Koo Lee

**Affiliations:** 1 Department of Preventive Medicine, Seoul National University College of Medicine, Seoul, Republic of Korea; 2 Cancer Research Institute, Seoul National University, Seoul, Republic of Korea; 3 Department of Biomedical Science, Seoul National University Graduate School, Seoul, Republic of Korea; 4 Department of Family Medicine, Seoul National University Hospital, Seoul, Republic of Korea; 5 Department of Family Medicine, Seoul National University College of Medicine, Seoul, Republic of Korea; 6 JW LEE Center for Global Medicine, Seoul National University College of Medicine, Seoul, Republic of Korea; 7 Seoul Center for Infectious Disease Control, Seoul, Republic of Korea

**Keywords:** Obesity; hypertension, Hyperglycemia, Dyslipidemia, Sensitivity and specificity, Area under the curve

## Abstract

**Background:**

Obesity is well known as a risk factor for cardiovascular disease. We aimed to determine the performance of and the optimal cutoff values for obesity indices to discriminate the presence of metabolic abnormalities as a primary risk factor for cardiovascular diseases in a Health Examinees study (HEXA).

**Methods:**

The current study analyzed 134,195 participants with complete anthropometric and laboratory information in a Health Examinees study, consisting of the Korean population aged 40 to 69 years. The presence of metabolic abnormality was defined as having at least one of the following: hypertension, hyperglycemia, or dyslipidemia. The area under the receiver operating characteristic curve (AUC) and 95% confidence intervals (CIs) were calculated for body mass index, waist to hip ratio, waist to height ratio, waist circumference, and conicity index.

**Results:**

The AUC of metabolic abnormalities was the highest for waist-to-height ratio (AUC [95% CIs], 0.677 [0.672–0.683] among men; 0.691 [0.687–0.694] among women), and the lowest for the C index (0.616 [0.611–0.622] among men; 0.645 [0.641–0.649] among women) among both men and women. The optimal cutoff values were 24.3 kg/m^2^ for the body mass index, 0.887 for the waist-to-hip ratio, 0.499 for the waist-to-height ratio, 84.4 cm for waist circumference and 1.20 m^3/2^/kg^1/2^ for the conicity index among men, and 23.4 kg/m^2^ for the body mass index, 0.832 for the waist-to-hip ratio, 0.496 for the waist-to-height ratio, 77.0 cm for the waist circumference and 1.18 m^3/2^/kg^1/2^ for the conicity index among women.

**Conclusion:**

The waist-to-height ratio is the best index to discriminate metabolic abnormalities among middle-aged Koreans. The optimal cutoff of obesity indices is lower than the international guidelines for obesity. It would be appropriate to use the indices for abdominal obesity rather than general obesity and to consider a lower level of body mass index and waist circumference than the current guidelines to determine obesity-related health problems in Koreans.

## Background

Obesity has emerged as a public concern worldwide, as its incidence has been steadily increasing [1]. The Global Burden of Disease Study reported that the estimated worldwide prevalence rates of obesity were 28.8 to 36.9% in men and 29.8 to 38.0% in women between 1980 and 2013 [2], and global death attributable to obesity rapidly increased more than doubling between 1990 and 2017 [3]. People with obesity are at increased risk for cardiovascular diseases, type 2 diabetes, certain cancers, and premature death [4, 5].

Body mass index is widely used for defining general obesity. The World Health Organization (WHO) recommended a lower cutoff point of body mass index for Asian populations than for Western populations [6], and it reflects the realization that adverse health is associated with a lower body mass index than the WHO criteria for Western countries [7]. Furthermore, recent studies have concluded that waist circumference, waist-to-hip ratio, and waist-to-height ratio better discriminate obesity-related metabolic abnormalities than body mass index [8–10]. Therefore, it is necessary to identify the optimal cutoff of obesity indices and to assess the discriminative power for obesity-related health problems in representative Asian populations. In this study, we aimed to evaluate the performance of obesity indices and to determine the optimal cutoff values for obesity indices to discriminate the presence of metabolic abnormalities in middle-aged Koreans. We comparatively investigated five obesity indices, including body mass index, waist circumference, waist-to-hip ratio, waist-to-height ratio, and conicity index, in the analyses.

## Method

### Study population

The Health Examinees (HEXA) study is a part of the Korea Genome Epidemiology Study (KoGES) [11]. National Health Insurance Corporation (NHIC) covers the entire Korean population for general health screening, and beneficiaries aged over 40 years can biannually receive screening through the national health examination program [12]. Participants in the HEXA study were prospectively recruited from 2004 to 2013 at 38 health examination centers and training hospitals located in 8 regions based on the infrastructural advantage of the national health checkup services funded by the Korea Centers for Disease Control and Prevention [11].

**Fig. 1:**
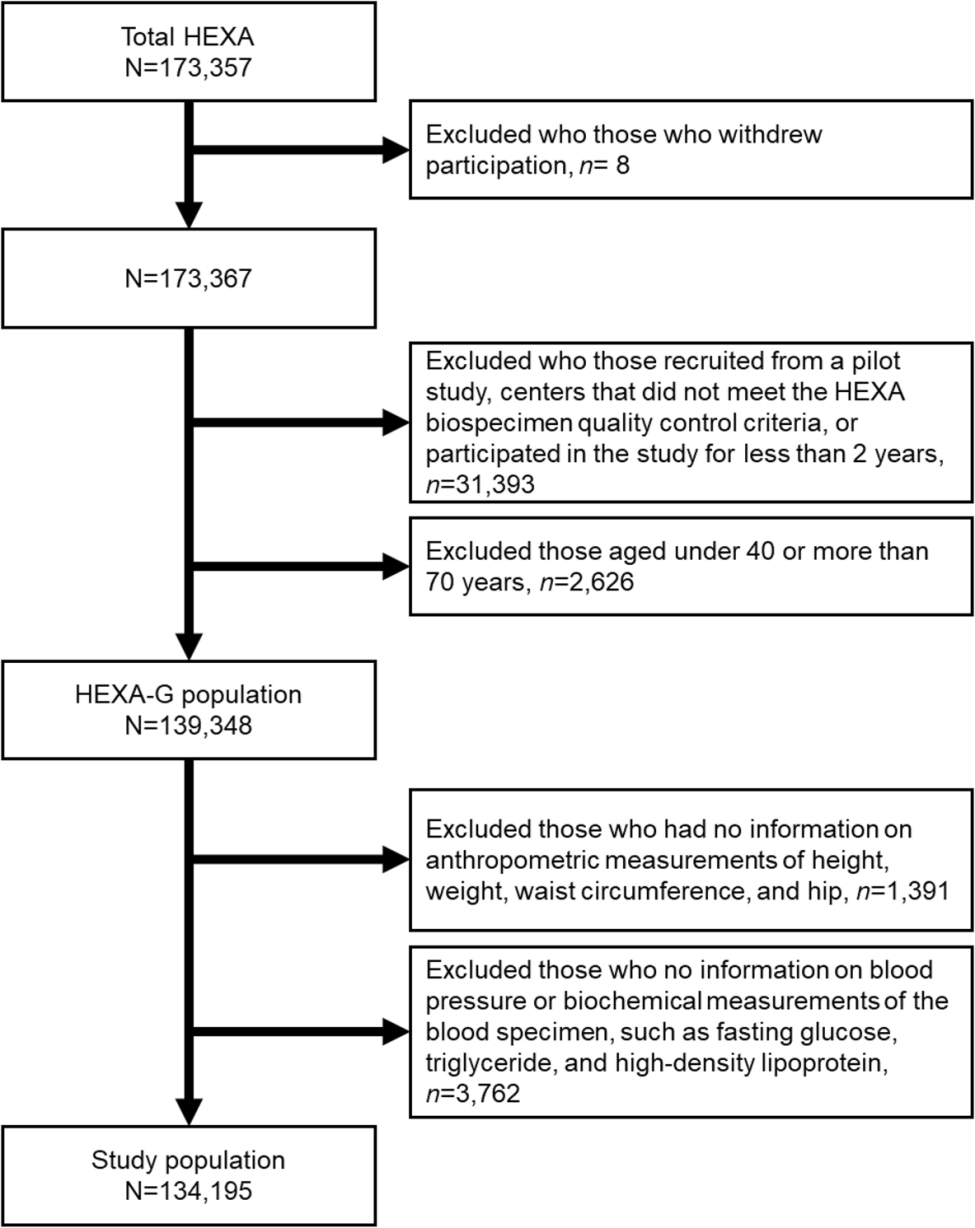
Inclusion and exclusion criteria of the study population

Figure 1 shows the flow chart of the study population. We included HEXA participants aged 40 to 69 years in the analyses and restricted them to Health Examinees-Gem (HEXA-G) participants who were defined as follows: we excluded (1) 8 sites (*n* = 9370) that only participated in the pilot study years 2004–2006, (2) 8 sites (*n* = 12,205) that did not meet the HEXA biospecimen quality control criteria (i.e., different testing protocols), and (3) 5 sites (*n* = 8799) that had participated in the study for less than 2 years. A total of 139,348 participants were included in the HEXA-G data. Among HEXA-G participants, we excluded 1391 participants who had no information on anthropometric measurements of height, weight, waist circumference, and hip circumference. An additional 3762 participants had no information on blood pressure or biochemical measurements of the blood specimen, such as fasting glucose, triglyceride, and high-density lipoprotein. We conducted all analyses among the 134,195 participants who remained after exclusion.

### Data collection

Participants were interviewed by trained interviewers and responded to a structured questionnaire on general characteristics and past medical history. Biochemical assessments and anthropometric measurements were also conducted for all participants. Blood specimens were taken after 8 h of fasting at enrollment and were transported to the clinical laboratory for blood tests using plasma to examine the levels of glucose, triglyceride, and high-density lipoprotein. Height was measured using digital freestanding stadiometers (BSM, InBody Co, Seoul, Korea) with the participants’ head in the Frankfort horizontal plane and was read up to one decimal place. Weight was measured using digital scales (BSM, InBody Co, Seoul, Korea) in units of 10 g. Waist and hip circumferences were obtained with a measuring tape in a horizontal plane and were read up to one decimal place. In detail, waist circumference was measured at the midpoint between the lower margin of the last palpable rib and the top of the iliac crest, and hip circumference was measured around the widest part of the buttocks.

Systolic and diastolic blood pressure values were manually measured using a stethoscope and mercury sphygmomanometer on one arm in the sitting position according to the standard operating procedure by trained medical staff. Blood pressure was measured at least twice, and the second blood pressure measurement was taken at least 1 min after the first measurement. If the difference between the two records of blood pressure was more than 5 mmHg, additional measurements were taken until the last two records of blood pressure were similar. Then, the last two records of blood pressures were recorded. Blood pressure was measured in both arms and was remeasured if the difference in blood pressure between both arms was more than 10 mmHg. Blood pressure was measured on the other arm only if there were arm injuries, previous breast surgery, venous or arterial tubes, or plaster bandages. We determined blood pressure as the average of the two readings.

### Definition of terms

Body mass index, waist circumference, waist-to-hip ratio, and waist-to-height ratio were calculated using directly measured anthropometric values. Additionally, we considered the conicity index as a measure of central adiposity with the equation below [13].

$$ Conicity\ index=\frac{waist\ circumference\ (m)}{0.109\times \sqrt{\frac{weight\ (kg)}{height\ (m)}}} $$

Participants meeting at least one of the following criteria were considered to have metabolic abnormalities: defined hypertension [14], those who had systolic blood pressure higher than 140 mmHg or diastolic blood pressure higher than 90 mmHg, or those who reported taking antihypertensive medication; hyperglycemia [15], those who had a fasting blood glucose higher than 126 mg/dL or who reported taking antidiabetic medication; and dyslipidemia, those who had a triglyceride level higher than 150 mg/mL, high-density lipoprotein cholesterol lower than 40 mg/dL, or those who reported taking medication for dyslipidemia. Low-density lipoprotein cholesterol is commonly used for diagnoses of dyslipidemia, but low-density lipoprotein cholesterol was not directly measured from blood samples. Therefore, we defined dyslipidemia using the levels of triglycerides and high-density lipoprotein cholesterol.

### Statistical analyses

We calculated the means and standard deviations for demographic, anthropometric, blood pressure, and biochemical characteristics. The inclusion of a large population in this study would reduce the meaningfulness of statistical significance for differences in the general characteristics between sexes. For this reason, we did not present a *p*-value in the descriptive analysis of Tables 1 and 2. Receiver operating characteristic (ROC) curves were plotted for obesity indices to identify the best obesity index that discriminates the presence of metabolic abnormalities. The area under the receiver operating characteristic curves (AUCs) was used as a summary measure of accuracy to evaluate the performance of obesity indices for the discrimination of participants with metabolic dysfunctions. Youden’s J statistics [16] were used to determine the optimal cutoff values for the obesity indices. Youden’s index was calculated using the equation below.

$$ {Youden}^{\prime }\ J\  statistics= sensitivity+ specificity-1 $$

**Table 1:** Anthropometric indices of obesity and metabolic characteristics among HEXA-G participants by sex, mean ± standard deviation

	Men (*N* = 45,052)	Women (*N* = 89,143)
Age, years	53.6 ± 8.38	52.3 ± 7.76
Height, cm	168.8 ± 5.74	156.5 ± 5.26
Weight, cm	69.6 ± 9.23	57.9 ± 7.66
Waist circumference, cm	85.7 ± 7.52	78.3 ± 8.17
Hip circumference, cm	96.0 ± 5.64	93.5 ± 5.69
Body mass index, kg/m^2^	24.4 ± 2.75	23.6 ± 2.94
Waist-to-hip ratio	0.89 ± 0.05	0.84 ± 0.06
Waist-to-height ratio	0.51 ± 0.04	0.50 ± 0.06
Conicity index, m^3/2^/kg^1/2^	1.23 ± 0.06	1.18 ± 0.08
Systolic blood pressure, mmHg	125.7 ± 14.35	120.6 ± 15.17
Diastolic blood pressure, mmHg	78.7 ± 9.68	74.7 ± 9.66
Fasting serum glucose level, mg/dL	99.3 ± 24.28	92.7 ± 18.47
Triglyceride, mg/dL	151.4 ± 111.41	112.5 ± 74.18
High-density lipoprotein cholesterol, mg/dL	49.4 ± 12.09	56.1 ± 12.80

**Table 2:** Prevalence of metabolic abnormalities among HEXA-G participants by sex

	Men	Women
Hypertension^a^, n (%)	15,310 (34.0)	21,386 (24.0)
Hyperglycemia^b^, n (%)	5196 (11.5)	5319 (6.0)
Dyslipidemia^c^, n (%)	21,794 (48.4)	39,768 (44.6)
Metabolic abnormalities^d^, n (%)	29,605 (65.7)	49,680 (55.7)

^a^Systolic blood pressure higher than 140 mmHg, diastolic blood pressure higher than 90 mmHg or those who reported taking antihypertensive medication

^b^Fasting glucose higher than 126 mg/dL or those who reported taking antidiabetic medication

^c^Triglyceride higher than 150 mg/mL, high-density lipoprotein cholesterol lower than 40 mg/dL or those who reported taking medication for dyslipidemia

^d^Having at least one of the aforementioned factors

We considered the optimal cutoff values at the corresponding value for the maximum Youden index. All statistical analyses were stratified by sex. We analyzed data using SAS version 9.4 (SAS Institute Inc., Cary, NC, USA) for the calculation of optimal cutoff points and R software version 3.6.3 [17] for the calculation of the AUCs.

## Results

The anthropometric indices of obesity and metabolic characteristics are presented in Table 1. The mean ages were 53.6 years for men and 52.3 years for women. Men had higher values than women for all anthropometric, blood pressure, and biochemical characteristics, except for high-density lipoprotein cholesterol. Table 2 describes the prevalence of hypertension, diabetes, hyperglycemia, dyslipidemia, and metabolic abnormalities among HEXA-G participants by sex. More than half of the participants had at least a single metabolic abnormality of diabetes, hypertension, or dyslipidemia in both men (65.7%) and women (55.7%). The prevalence of metabolic abnormalities was higher in men than in women. Among the components of metabolic abnormalities, the prevalence was the lowest for hyperglycemia (11.5% for men; 6.0% for women) and highest for dyslipidemia (48.4% for men; 44.6% for women).

The AUCs of the obesity indices associated with metabolic abnormalities and their components are shown in Table 3. Among men, the highest AUCs to discriminate metabolic abnormalities were obtained in the waist-to-height ratio (AUC [95% confidence intervals], 0.677 [0.672–0.683]), followed by the waist circumference (0.671 [0.666–0.677]), body mass index (0.667 [0.661–0.672]) and the waist-to-hip ratio (0.656 [0.650–0.661]) and conicity index (0.616 [0.611–0.622]); the AUCs of the waist-to-hip ratio and the conicity index were significantly lower than those of the waist-to-height ratio, the waist circumference, and the body mass index. Among women, the highest AUCs were obtained in the waist-to-height ratio (0.691 [0.687–0.694]), followed by the waist-to-hip ratio (0.681 [0.677–0.684]), the waist circumference (0.680 [0.677–0.684]), the body mass index (0.668 [0.665–0.672]) and the conicity index (0.645 [0.641–0.649]). Women had higher AUCs associated with metabolic abnormalities for all obesity indices than men.

**Table 3:** Areas under the receiver operating characteristic curves and the corresponding 95% confidence intervals of obesity indices associated with metabolic abnormalities and their components among HEXA-G participants by sex

	Men	Women
*Hypertension*^a^		
Body mass index	0.629 (0.624–0.635)	0.668 (0.664–0.672)
Waist circumference	0.629 (0.624–0.635)	0.671 (0.667–0.675)
Waist-to-hip ratio	0.617 (0.612–0.623)	0.665 (0.661–0.669)
Waist-to-height ratio	0.646 (0.640–0.651)	0.687 (0.683–0.691)
Conicity index	0.593 (0.587–0.598)	0.635 (0.631–0.639)
*Hyperglycemia*^b^		
Body mass index	0.570 (0.562–0.579)	0.642 (0.634–0.649)
Waist circumference	0.605 (0.597–0.613)	0.683 (0.675–0.690)
Waist-to-hip ratio	0.636 (0.629–0.644)	0.715 (0.708–0.721)
Waist-to-height ratio	0.616 (0.608–0.624)	0.693 (0.686–0.700)
Conicity index	0.611 (0.603–0.619)	0.674 (0.667–0.681)
*Dyslipidemia*^c^		
Body mass index	0.645 (0.640–0.650)	0.636 (0.632–0.639)
Waist circumference	0.645 (0.640–0.651)	0.648 (0.644–0.652)
Waist-to-hip ratio	0.625 (0.620–0.630)	0.651 (0.647–0.654)
Waist-to-height ratio	0.641 (0.636–0.646)	0.653 (0.649–0.657)
Conicity index	0.587 (0.582–0.593)	0.618 (0.614–0.622)
*Metabolic abnormalities*^d^		
Body mass index	0.667 (0.661–0.672)	0.668 (0.665–0.672)
Waist circumference	0.671 (0.666–0.677)	0.680 (0.677–0.684)
Waist-to-hip ratio	0.656 (0.650–0.661)	0.681 (0.677–0.684)
Waist-to-height ratio	0.677 (0.672–0.683)	0.691 (0.687–0.694)
Conicity index	0.616 (0.611–0.622)	0.645 (0.641–0.649)

^a^Systolic blood pressure higher than 140 mmHg, diastolic blood pressure higher than 90 mmHg or those who reported taking antihypertensive medication

^b^fasting glucose higher than 126 mg/dL or those who reported taking antidiabetic medication

^c^triglyceride higher than 150 mg/mL, high-density lipoprotein cholesterol lower than 40 mg/dL or those who reported taking medication for dyslipidemia

^d^Having at least one of the aforementioned factors

Table 4 shows the optimal cutoff values of obesity indices for metabolic abnormalities. Optimal cutoff values to discriminate metabolic abnormalities were 24.3 kg/m^2^ for the body mass index, 84.4 cm for the waist circumference, 0.887 for the waist-to-hip ratio, 0.499 for the waist-to-height ratio and 1.20 m^3/2^/kg^1/2^ for the conicity index for men and 23.4 kg/m^2^ for the body mass index, 77.0 cm for the waist circumference, 0.832 for the waist-to-hip ratio, 0.496 for the waist-to-height ratio and 1.18 m^3/2^/kg^1/2^ for the conicity index for women.

**Table 4:** Optimal cutoff values, Youden indices, and sensitivity and specificity of obesity indices associated with metabolic abnormalities and their components among HEXA-G participants by sex

	Men				Women			
	Optimal cutoff value	Youden’s index	Sensitivity	Specificity	Optimal cutoff value	Youden’s index	Sensitivity	Specificity
*Hypertension*^a^								
Body mass index	24.5	0.189	0.596	0.593	23.5	0.246	0.670	0.576
Waist circumference	87.0	0.187	0.570	0.617	78.9	0.255	0.655	0.600
Waist-to-hip ratio	0.896	0.175	0.594	0.581	0.840	0.241	0.662	0.580
Waist-to-height ratio	0.506	0.210	0.662	0.548	0.503	0.275	0.674	0.601
Conicity index	1.24	0.138	0.535	0.604	1.19	0.199	0.607	0.592
*Hyperglycemia*^b^								
Body mass index	24.5	0.104	0.567	0.537	24.2	0.207	0.569	0.637
Waist circumference	86.3	0.150	0.591	0.559	80.0	0.272	0.672	0.600
Waist-to-hip ratio	0.895	0.197	0.660	0.537	0.850	0.315	0.710	0.606
Waist-to-height ratio	0.509	0.165	0.634	0.531	0.514	0.288	0.655	0.633
Conicity index	1.23	0.165	0.655	0.510	1.18	0.255	0.728	0.527
*Dyslipidemia*^c^								
Body mass index	24.0	0.213	0.654	0.559	23.2	0.198	0.634	0.564
Waist circumference	84.4	0.208	0.675	0.533	77.5	0.219	0.640	0.579
Waist-to-hip ratio	0.879	0.185	0.712	0.474	0.832	0.227	0.652	0.575
Waist-to-height ratio	0.502	0.204	0.661	0.542	0.497	0.228	0.636	0.592
Conicity index	1.20	0.131	0.706	0.425	1.18	0.175	0.608	0.567
*Metabolic abnormality*^d^								
Body mass index	24.3	0.241	0.588	0.653	23.4	0.245	0.604	0.641
Waist circumference	84.4	0.248	0.653	0.595	77.0	0.266	0.676	0.590
Waist-to-hip ratio	0.887	0.228	0.639	0.589	0.832	0.270	0.645	0.624
Waist-to-height ratio	0.499	0.259	0.672	0.587	0.496	0.281	0.638	0.644
Conicity index	1.20	0.173	0.698	0.475	1.18	0.210	0.598	0.613

^a^Systolic blood pressure higher than 140 mmHg, diastolic blood pressure higher than 90 mmHg or those who reported taking antihypertensive medication

^b^Fasting glucose higher than 126 mg/dL or those who reported taking antidiabetic medication

^c^Triglyceride higher than 150 mg/mL, high-density lipoprotein cholesterol lower than 40 mg/dL or those who reported taking medication for dyslipidemia

^d^Having at least one of the aforementioned factors

## Discussion

Using the baseline data from a large community-based cohort study, we examined the discriminative performance of obesity indices for the presence of metabolic abnormalities by calculating the AUCs. The highest discriminative power to discriminate metabolic abnormalities was shown for the waist-to-height ratio among both men and women. Compared with body mass index, as an index of general obesity, indices for abdominal obesity (i.e., waist circumference, waist-to-hip ratio and waist-to-height ratio) show better performance in discriminating abnormal metabolic status. Among the indices for abdominal obesity, there was no significant difference in the discriminative power to the presence of metabolic abnormalities between waist circumference and waist-to-hip ratio.

We also determined the optimal cutoff values of obesity indices in the present study. The optimal cutoff of the waist-to-height ratio for all factors of metabolic abnormalities was approximately 0.5 for both men and women, which is consistent with the results from a previously published systematic review [18]. The optimal cutoff values of the waist circumference, waist-to-hip ratio and body mass index were different for each factor of metabolic abnormalities.

Although body mass index is widely used to determine healthy weight, abdominal obesity indices have been reported to be better tools to discriminate the presence of metabolic abnormalities in Asians [19–23], Australians [24], and Americans [25]. A previous prospective cohort study on the obesity index and metabolic dysfunction also showed that abdominal obesity indices, especially the waist-to-hip ratio, were a better predictor than body mass index for developing multiple metabolic risk factors in the Korean population [26].

A meta-analysis from previously published cross-sectional or cohort studies compared the AUCs of waist-to-height ratio, waist circumference and body mass index in discriminating obesity-related disease and concluded that the waist-to-height ratio has superior performance over body mass index and waist circumference [8]. A higher discriminative power of the waist-to-height ratio than body mass index was also presented in other meta-analysis results based on previously conducted prospective studies [9]. Furthermore, the waist-to-height ratio was shown to be a significantly better tool than waist circumference in a meta-analysis of within-study differences in AUCs between the waist-to-height ratio and waist circumference [8]. However, the difference in the discriminative performance of body mass index, waist circumference and waist-to-hip ratio has not shown statistical significance in the pooled analysis of over eighty thousand individual data from British cohorts [10].

The optimal cutoff values of body mass index and waist circumference from the present study were lower than previous guidelines of the WHO [6] and the National Cholesterol Educational Program (NCEP) [27]. Our results showed that body mass indices of more than 24.3 kg/m^2^ among men and 23.4 kg/m^2^ among women were associated with the presence of metabolic abnormalities, while the WHO recommended that obesity in Asian populations be defined as a body mass index of 25 kg/m^2^ or higher [6]. We obtained waist circumference cutoffs of 84.4 cm for men and 77.0 cm for women, and these results are lower than the NCEP guidelines for metabolic syndrome, which defined obesity as a waist circumference of more than 90 cm for men and 80 cm for women [9]. We suggested that a lower cutoff abdominal obesity index could discriminate metabolic dysfunction in Asians. A previous prospective cohort study in Korea also described that lower waist circumference (80 cm for men and 78 cm for women) had a better performance to predict the development of metabolic abnormalities than the NCEP guidelines [27].

This study had several limitations. First, there is an inherent limitation of temporality in the cross-sectional design. However, our results are similar to the results from prospective studies in Korea [20, 26], and we suggest that the theoretical effect of the temporality of our results is not strong. Second, it is difficult to generalize these results to young people. The study participants consisted of Koreans aged 40 to 60 years who regularly attended health screening examinations; therefore, the cutoff values derived from the current study may be relevant to the middle-aged Korean population only.

## Conclusions

The waist-to-height ratio is the best index to discriminate metabolic abnormalities among middle-aged Koreans. The optimal cutoff of obesity indices of body mass index and waist to circumference is lower than the recommendations from the WHO and NCEP. Based on these findings, to determine obesity-related health problems in Koreans, it would be appropriate to use indices for abdominal obesity rather than general obesity and to consider a lower level of body mass index and waist circumference than the current guidelines. We hope that these results will improve the guidelines for screening populations at high risk for cardiometabolic diseases via appropriate recommendations for obesity in Korea.

## Data Availability

The datasets generated and analyzed during the current study are not publicly available due to ethical considerations of the participants’ confidentiality but are available from the corresponding author upon reasonable request.

## Abbreviations

AUC: Area under the receiver operating characteristic curve
WHO: World Health Organization
KoGES: The Korea Genome Epidemiology Study
HEXA: Health Examinees in KoGES
HEXA-G: Health Examinees-Gem in KoGES
ROC: Receiver operating characteristic
